# Effects of Density Stress During Transportation on the Antioxidant Activity and Immuno-Related Gene Expression in Yellowfin Seabream (*Acanthopagrus latus* Houttuyn, 1782)

**DOI:** 10.3390/genes15111479

**Published:** 2024-11-17

**Authors:** Xiulin Nong, Kecheng Zhu, Huayang Guo, Baosuo Liu, Nan Zhang, Qin Zhang, Dianchang Zhang

**Affiliations:** 1 Guangxi Marine Microbial Resources Industrialization Engineering Technology Research Center, Guangxi Key Laboratory for Polysaccharide Materials and Modifications, School of Marine Sciences and Biotechnology, Guangxi Minzu University, 158 University Road, Nanning 530008, China; nxiulin@163.com; 2 Key Laboratory of South China Sea Fishery Resources Exploitation and Utilization, Ministry of Agriculture and Rural Affairs, South China Sea Fisheries Research Institute, Chinese Academy of Fishery Sciences, Guangzhou 510300, China; zkc537@163.com (K.Z.); guohuayang198768@163.com (H.G.); liubaosuo343@163.com (B.L.); 398730316@163.com (N.Z.); 3 Guangdong Provincial Engineer Technology Research Center of Marine Biological Seed Industry, Guangzhou 510300, China; 4 Sanya Tropical Fisheries Research Institute, Sanya 572018, China

**Keywords:** antioxidant, transportation, density stress, gene expression, *Acanthopagrus latus*

## Abstract

**Background/Objectives:** Maintaining an optimum transport density is essential for protecting water quality, lowering stress levels, and increasing fish survival rates. Transporting marine fish fry involves major dangers. The purpose of this study was to evaluate the impact of transport stress at varying densities on the immune-related gene expression, antioxidant capacity, and survival rate of yellowfin seabream (*Acanthopagrus latus*) fry. **Methods:** A 12 h simulated transport experiment was conducted with *A. latus* fry divided into six density groups. For 1–2 cm fry, densities of 900, 1200, and 1500 fry per pouch were used to assess antioxidant enzyme activity; and for 4–5 cm fry, densities of 100, 125, and 150 fry per pouch were used for gene expression analysis. The key parameters measured included survival rates, antioxidant enzyme activities in liver and intestinal tissues, and expression levels of *HSP90α* and *caspase-3* genes. **Results:** The findings showed that recovery time and density both affected the observed responses and that transport density had a substantial effect on antioxidant enzyme activity in all tissues. The intestinal and liver tissues showed a considerable decrease in antioxidant enzyme activity, suggesting that these tissues may be able to respond to oxidative stress. Moreover, under high-density transport conditions, there were notable increases in the expression of *caspase-3* and *HSP90α*, suggesting the activation of immune response systems. This research offers valuable new understandings into the relationship between transport density and immunological and antioxidant modulation in *A. latus* fry. **Conclusions:** The results provide a scientific foundation for enhancing aquaculture transport conditions, which will ultimately lead to decreased fish mortality and improved general health during transit, resulting in more sustainable and effective aquaculture methods.

## 1. Introduction

The transportation of fish fry is a crucial and essential aspect of aquaculture and often induces stress in fish, primarily due to handling and transportation procedures. Environmental stress, which refers to the pressure exerted by the external environment on the well-being of aquatic animals, can lead to stress and damage to fry during transportation [1,2,3]. Confined transport is commonly utilized in aquaculture, as it reduces the risk of disease transmission. In confined transport, fish are provided with an additional oxygen supply by injecting gas or air directly into the fish bags before transportation [4]. However, this process does not prevent the rapid acidification of the medium, caused by the sharp increase in carbon dioxide (CO_2_) resulting from fish respiration, ammonia accumulation, or other factors that may adversely affect fish homeostasis during and after transportation [5,6].

Fish are susceptible to oxidative stress induced by ROS and have developed antioxidant defense mechanisms characterized by the presence of specific adaptive enzymes, including CAT, POD, and T-SOD. T-SOD, POD, and CAT are essential enzymes involved in the elimination of ROS and serve as critical components in mitigating the harmful effects of oxidative stress [7]. T-SOD catalyzes the conversion of superoxide radicals into oxygen or hydrogen peroxide, which CAT then converts into water and oxygen [8]. LDH, linked to cellular metabolism, is essential in anaerobic fermentation and gluconeogenesis, playing a key role in various metabolic pathways [9]. These enzymes have been detected in the majority of fish species investigated to date [10,11]. Peng et al. (2014) showed that after continuous transport for 6 h, catalase (CAT) and superoxide dismutase (SOD) activities in the livers of *Nibea japonica* increased rapidly and were significantly higher than those before transportation [12]. The activities then gradually decreased to normal levels, confirming that SOD and CAT could be used as effective bioindicators for the detection of stress. Liu et al. (2018) investigated the effects of oxygenated transport in a closed plastic bag on the activity of SOD, CAT, and other enzymes and the total antioxidant capacity in juvenile four-fingered horse mackerel (*Eleutheronema tetradactylum*) [13]. The results showed that the fish suffered significant stress and that the antioxidant system was affected by transportation stress. Bai showed that the density stress of juvenile Pelteobagrus fulvidraco during simulated transportation was associated with the finding that as transport time increased, CAT levels in the liver initially rose and then decreased after 16 h, while SOD activity continued to increase [14]. This confirmed that density causes stress to fish during transport.

Regulation of hypoxia-inducible factor 1-alpha (*HIF-1α*) expression and activity can occur through a variety of mechanisms, including altered mRNA expression, protein expression, and transcriptional activation [15]. Heat shock protein 90 (*HSP90α*) is involved in the repair of proteins that have been damaged by stress, promoting normal cell growth by activating protein synthesis, and assisting in the refolding of new proteins [16]. As a member of the cysteine protease family, *caspase-3* is involved in cellular immunity by transmitting external signals into the cell, ultimately causing apoptosis [17]. The multifunctional cytokine interleukin *(IL)-6* is crucial for the signaling and coordination of both innate and acquired immune responses [18]. Hong found that when Asian sea bass fry were subjected to different levels of transport stress, cytokines were activated to promote inflammation, heat shock protein genes were upregulated, and HSP90 exerted its reparative capacity only when the transport time exceeded 8 h [19]. The results showed that under transportation stress, the stress response of fish was significant and antioxidant genes were affected.

*A. latus* belongs to the seabream family and is a saline bony fish widely distributed in the southeastern saltwater of China. Its meat is delicious, and it is an important economic fish [20,21]. Due to its favorable taste profile and high nutritional value, *A. latus* is highly demanded in the market. This species holds significant value in fisheries and shows great potential for aquaculture. Research indicates that enhancing growth, reproduction, and health management in *A. latus* can improve aquaculture efficiency and reduce disease incidence and mortality rates [22]. Studies have investigated the immune genes related to disease resistance in *A. latus* fry under transport density stress [23]. With the development of the aquaculture industry, the demand for *A. latus* fry is increasing; therefore, it is crucial to explore the effect of transport density on their survival rate and antioxidant capacity, which are essential to ensure the quality of fry during transportation. To date, few studies have been conducted on stress in *A. latus* fry. In this study, we examined the changes in the expression of relevant immune genes and antioxidant enzyme activities in various *A. latus* fry tissues at different transport densities and explored suitable conditions for *A. latus* fry under transport density stress. Our results provide a scientific basis for the transport of *A. latus* fry.

## 2. Materials and Methods

### 2.1. Experimental Material

Prior to the experiment, *A. latus* fry 1–2 cm and 4–5 cm in length were selected from healthy, active, wild individuals that showed no signs of injury. The size of the plastic bags used in the experiment was 48 × 37 × 22.5 cm. Each plastic bag was filled with 4400 mL of seawater from a nursery pool at a temperature of 20 °C, salinity of 32%, and pH of 7.90, and then sealed after oxygenation.

### 2.2. Simulated Transportation Experiment

The experiment was performed using the method described by Liang et al. (2023) [23]. *A. latus* fry ranging from 1–2 cm in size were allocated to three density groups (D1 = 900 fry per pouch, D2 = 1200 fry per pouch, and D3 = 1500 fry per pouch), with three pouches per density group, totaling 10,800 samples for evaluating antioxidant enzyme activities. Similarly, *A. latus* fry measuring 4–5 cm in size were segregated into three density groups (D4 = 100 fry per pouch, D5 = 125 fry per pouch, and D6 = 150 fry per pouch), also with three pouches per density group, totaling 1125 samples for assessing antioxidant enzyme-related gene expression. After a 12 h transportation test, the effect of varying recovery durations (0, 6, 12, and 18 h) on density-induced stress was investigated. The recovery time at 0 h served as the only control group, and there were three replicates for each group, in accordance with standard scientific protocols.

### 2.3. Sample Collection and Analysis

The samples obtained at 0 h were used as controls. Water and whole-fish samples were collected at 0, 6, 12, and 18 h after transportation, and the mortality rates of the various groups were determined. Total SOD (T-SOD), lactate dehydrogenase (LDH), catalase (CAT), and peroxidase (POD) activities in the liver were analyzed using a kit developed by the Nanjing Jiancheng Bioengineering Institute (Nanjing, China). Whole fish were dissected and the relevant tissues were snap-frozen in liquid nitrogen tanks; RNAlater was used for gene expression analysis.

### 2.4. Total RNA Extraction and Reverse Transcription

Total RNA was extracted from fish liver, intestines, muscle, gills, and whole fish using the HiPure Universal RNA Mini Kit (Guangzhou Magen Biotechnology, Guangzhou, China) and Ambion RNAlater solution (Thermo Fisher Scientific, Waltham, MA, USA) according to the manufacturer’s instructions. The quality and concentration of the RNA samples were assessed using 1.2% agarose gel electrophoresis and a NanoDrop™ 2000 spectrophotometer (Thermo Fisher Scientific). cDNA was produced using a cDNA synthesis kit (Accurate Biotechnology, Changsha, China), according to the manufacturer’s instructions, and used in subsequent real-time RT-PCR analysis.

### 2.5. Whole-Fish Gene Expression

The cDNA sequences of *HIF-1α*, *HSP90α*, *caspase-3*, and *IL-6* genes were obtained from the chromosome-level gene assemblies of cDNA sequences for *A. latus*-related genes provided by our research group (Table 1). Primer design was performed using Oligo 7 software (Colorado Springs, CO, USA). SYBR Green Pro Taq HS premix (Hunan Accurate Biotechnology Co., Ltd., Changsha, China) and the Roche Light Cycler 480 (Basel, Switzerland) were used to determine the relative expression of the selected genes according to the manufacturer’s instructions. β-actin was used as the reference gene. Single peaks were observed in the melting curve analysis, indicating a single PCR product and ensuring that there were no non-specific products at the end of each PCR. The 2^−ΔΔCt^ technique was used to calculate the relative expression levels.

### 2.6. Statistical Analysis

All statistical analyses were performed using SPSS version 23.0 (IBM Corp., Armonk, NY, USA). All information is presented as the standard deviation (mean ± SE), and subsequently, differences between groups were analyzed using one-way and two-way analysis of variance, followed by Duncan’s multiple range test. The minimum level of significance was set at 95% (*p* < 0.05).

## 3. Results

### 3.1. Effect of Transportation Density on the Survival of Juvenile A. latus

After 12 h of simulated transportation, the survival rates of *A. latus* fry in the D1 to D6 groups were 100%. The fry was then placed in a recirculating water system, where they demonstrated normal swimming behavior and stable respiration rates during an 18 h recovery period. After this period of recovery, the survival rate across all density groups remained at 100%.

### 3.2. Changes in Antioxidant Capacity Enzyme Activities in Different Tissues

**Figure 1:**
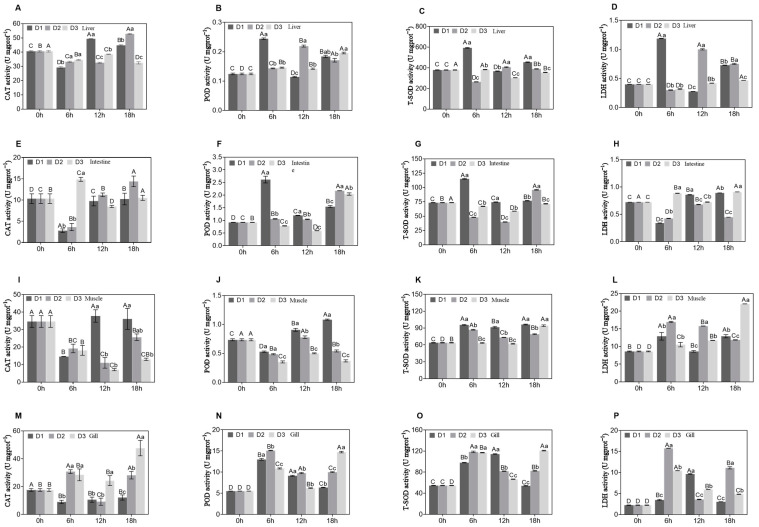
The activities of antioxidant enzymes CAT, POD, T-SOD, and LDH in *A. latus* liver (**A**–**D**), intestine (**E**–**H**), muscle (**I**–**L**), and gill (**M**–**P**) under different density stresses (D1, D2, D3) after 12 h transportation (6 h, 12 h, and 18 h sampling time points). The 0 h group served as the control group. Different letters on the bar graph indicate significant differences (*p* < 0.05).

In the liver tissue (Figure 1A–D), CAT activity increased with prolonged recovery time, particularly in the high-density (D3) group. POD activity was initially lower at 0 h but significantly increased after 6 h, especially in the D2 and D3 groups (*p* < 0.05). T-SOD activity peaked at 6 h and subsequently declined but increased at 18 h in the D3 group. LDH activity showed considerable fluctuations across various densities and time points, with the highest activity recorded at 18 h in the low-density (D1) group, whereas in the D3 group, it peaked at 6 h. In summary, density and post-transport recovery time significantly influenced the enzymatic activities of CAT, POD, T-SOD, and LDH in the liver tract (*p* < 0.05). Furthermore, a statistically significant interaction between density and recovery time was observed (*p* < 0.05).

In the intestinal tissue (Figure 1E–H), CAT activity showed an overall increasing trend with prolonged recovery time, which was notably pronounced in the D3 group. POD activity was initially low at 0 h and significantly increased after 6 h in the D2 and D3 groups. T-SOD activity peaked at 6 h and subsequently declined but increased at 18 h in the D3 group. LDH activity demonstrated considerable fluctuations across various densities and time points, with the highest activity recorded at 18 h in the D1 group, whereas in the D3 group, it peaked at 6 h. In summary, density and post-transport recovery time significantly influenced the enzymatic activities of CAT, POD, T-SOD, and LDH in the intestinal tract (*p* < 0.05). Furthermore, a statistically significant interaction was observed between density and recovery time (*p* > 0.05).

In muscle tissues, the activities of the CAT, POD, and T-SOD (Figure 1I–L) enzymes initially increased, then stabilized or slightly decreased, reaching peak levels 18 h post-stress. The LDH enzyme activity exhibited variable trends at different recovery times; however, elevated activity levels were observed after 18 h of recovery. The effect of varying density stress on enzymatic activity was statistically significant (*p* < 0.05). Furthermore, a statistically significant interaction was observed between density and recovery time (*p* < 0.05).

In the gill tissues (Figure 1M–P), CAT activity showed an increasing trend under all density conditions as the recovery time increased, with a particularly significant increase observed at 18 h in the D3 group (*p* < 0.05). POD activity was significantly increased at 6 h post-recovery in the D1 and D2 groups (*p* < 0.05), whereas at 12 and 18 h, the activity was significantly higher in the D3 group compared with the other groups (*p* < 0.05). T-SOD activity was significantly higher in the D2 group at both 6 h and 18 h compared with the other groups (*p* < 0.05). LDH activity was significantly increased across all density conditions at the onset of recovery (6 h post-recovery), with the increase being especially pronounced in the D2 group (*p* < 0.05).

**Figure 2:**
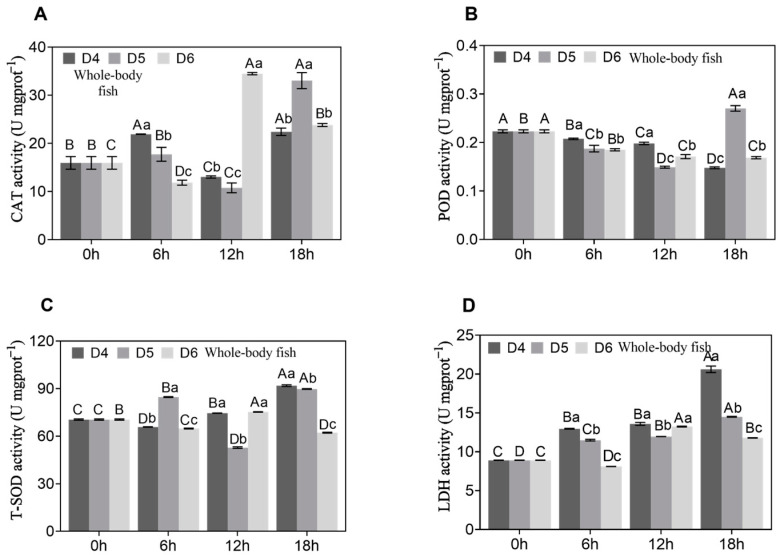
Antioxidant enzyme activity of CAT (**A**), POD (**B**), T-SOD (**C**), and LDH (**D**) in whole fish under different density stresses (D4, D5, D6) after 12 h transportation (6 h, 12 h, and 18 h sampling time points). The 0 h group served as the control group. Different letters on the bar graph indicate significant differences (*p* < 0.05).

In the whole-fish samples, CAT activity was relatively reduced at the initial time point (0 h) following transport density stress (Figure 2). As the recovery period progressed, a gradual increase in CAT activity was observed, culminating in peak levels at 12 h and 18 h (*p* < 0.05). The activity of POD remained consistent during the early recovery phase but exhibited a significant increase during the later stages (*p* < 0.05). Similarly, T-SOD activity, which was initially low, showed a significant increase over the recovery period (*p* < 0.05), reaching its peak at 18 h. LDH activity was the lowest at the initial time point of 0 h and then gradually increased, peaking at 18 h.

### 3.3. Effect of Transport Density on the Expression of Immune Genes

The gene expression profiles of *HIF-1α*, *HSP90α*, *caspase-3*, and *IL-6* were assessed across 13 normal dissection tissues of *A. latus* fry, including the liver, spleen, intestines, stomach, kidneys, heart, brain, eyes, muscles, skin, gills, fins, and gonads (Supplement S1). Liver tissue was used as a benchmark group to investigate relative mRNA expression levels. All tissues showed expression of these target genes. In the non-experimental group, the highest expression of *HIF-1α* was observed in the intestine, whereas the lowest expression was in the fins. *HSP90α* showed the highest expression in the intestine and the lowest in the muscles. *IL6* showed the highest expression in the skin. *Caspase-3* showed the highest expression in the muscles, followed by the kidneys, with the lowest expression observed in the fins.

**Figure 3:**
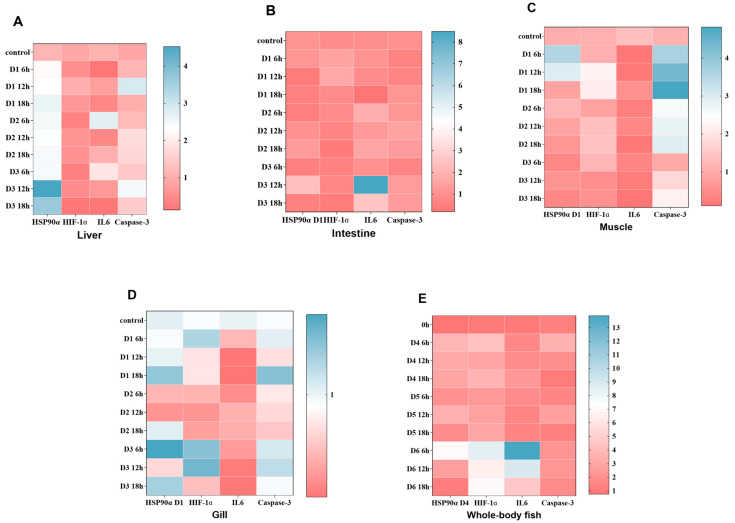
The relative expressions of immune-related genes in *A. latus* fry liver (**A**), intestine (**B**), muscle (**C**), gill (**D**), and whole-body fish (**E**) under different density stresses (D1, D2, D3, D4, D5, D6) after 12 h transportation (6 h, 12 h, and 18 h sampling time points). The 0 h group was the control group. Heatmap was constructed by Graphpad Prism 8 software.

In liver tissue (Figure 3A), *HSP90α* expression was higher than that of the other two density groups in the D3 group, especially at the 12 h and 18 h recovery time points. The expression level of *HIF-1α* did not vary significantly across different density groups and recovery times; however, there was a slight decrease at the 18 h recovery time point in the D2 group. *IL-6* showed a significant increase in expression at the 6 h recovery time point in the D1 group and a decrease in expression at the 18 h recovery time point in the D2 and D3 groups. The expression of *caspase-3* increased with recovery time in the D1 and D2 groups, whereas it was lower at the 12 h and 18 h recovery time points in the D3 group.

In the intestinal tissue (Figure 3B), the expression of *HSP90α*, *HIF-1α*, and *caspase-3* remained consistently high across all density groups and recovery time points. *IL-6* also exhibited relatively high expression levels in the control group and in most combinations of density and recovery time, with a significant decrease observed at the 12 h recovery time point in the D3 group (*p* < 0.05).

In muscle tissue (Figure 3C), the expression level of *HSP90α* at the 6 h recovery time point in the D1 group decreased compared to the control group and gradually increased at the 12 and 18 h recovery time points. In the D2 and D3 groups, gene expression was relatively stable at all time points, with no significant differences compared with the control group. The expression of *HIF-1α* at 6 h in the D1 group was significantly increased compared to the control (*p* < 0.05). The expression of *IL-6* in the D1 group was lower at 6 h but significantly increased at 12 and 18 h (*p* < 0.05). In the D1 group, *caspase-3* significantly increased at 12 and 18 h (*p* < 0.05). In the D2 and D3 groups, the expression level decreased at 6 h but did not show a significant increase at 12 h and 18 h.

In gill tissue (Figure 3D), the expression of *HSP90α* increased with recovery time in D1, decreased after 6 h, and then increased again in D2 and D3. *HIF-1α* exhibited higher expression levels in the D1 group at 6 h, which then gradually decreased. In the D2 and D3 groups, *HIF-1α* maintained a relatively stable expression throughout the recovery process. *IL-6* showed an increasing trend in expression during the recovery period across all density groups, particularly in D1, where it peaked at 18 h. *Caspase-3* showed a significant increase in expression (*p* < 0.05) in D1 after 6 h of recovery, followed by a decrease. In the D2 and D3 groups, the expression of caspase-3 did not vary significantly during recovery.

In the whole-fish samples (Figure 3E), the expression of *HSP90α* in the D4 group decreased with increasing recovery time and reached a relatively low level at 18 h. The expression of *HIF-1α* in the D4 group peaked at 6 h of recovery and then declined at 12 and 18 h of recovery. The expression of *IL-6* decreased significantly after 18 h of recovery in the D5 group (*p* < 0.05). The expression of *caspase-3* was stable across all density groups and recovery time points, with no significant trends.

## 4. Discussion

The present study identified a significant modulation of CAT, POD, T-SOD, and LDH activity across various tissues of *A. latus* fry, such as intestine, muscle, gill, and whole body, in response to transport stress under different density conditions. CAT activity in the liver of the D3 group was significantly increased, indicating an enhanced response to oxidative stress in the fish. This finding aligns with Livingstone’s (2003) study, which demonstrated that aquatic animals respond to oxidative damage by increasing CAT activity [24]. The activity of POD initially decreased and subsequently increased dramatically, particularly in the D2 and D3 groups, suggesting a delayed reaction to oxidative stress. T-SOD activity in the D3 group reached its highest point after 6 h, then decreased and further spiked after 18 h. This pattern is consistent with the findings of Shi et al. (2022) regarding T-SOD activity in stressed fish. T-SOD plays a crucial role in the imbalance of superoxide radicals, and its activity pattern indicates an adaptive reaction to the initial phase of stress, followed by recuperation [25]. T-SOD activity in the fish in all the D1 to D3 groups was lower than in the control group. This decrease could be related to the buildup of a significant number of free radicals in the fish under high-density conditions. This accumulation can lead to damage and decreased immunity [14]. LDH activity reached its maximum level at 18 h in the D1 group and peaked at 6 h in the D3 group. This suggests that transportation stress affected the energy metabolism rate of the fish, and the significant increase in enzyme activity may reflect structural changes in the liver and other tissues under stress conditions. This observation is consistent with previous research patterns on metabolic changes in fish exposed to high-density environments [26].

In the intestinal tissue, the D3 group exhibited the highest antioxidant enzyme activity, suggesting that under high-density conditions, fish may regulate oxidative stress by increasing the quantity of antioxidant enzymes to alleviate oxidative damage in the intestine and other tissues [27]. This finding is consistent with results from a high-density farming experiment on young largemouth bass, which also observed a significant increase in intestinal antioxidant enzyme activity under high-density conditions [28]. High-density environments are often accompanied by the accumulation of metabolic waste and the deterioration of water quality, increasing the risk of oxidative stress. Therefore, the enhancement of antioxidant enzymes may be a key adaptive mechanism for fish in such environments, helping to maintain intestinal health and reduce stress-induced inflammatory responses [29].

In the muscle tissues, the level of LDH enzyme activity increased after 18 h of recovery, and the CAT, POD, and T-SOD enzyme activities showed the same trend. This finding aligns with Zhu’s (2022) study, which demonstrated that anaerobic respiration capacity is enhanced while aerobic respiration capacity is reduced during this process [30]. Tenji suggested that the synergistic increase in enzyme activity may be an adaptive defense mechanism in fry against unfavorable environments [31]. Under different nutritional conditions, the muscle tissues of *A. latus* maintain tissue integrity and health by regulating their metabolic and antioxidant processes [32]. The increase in antioxidant enzyme activity in this study suggests that muscle tissue activates defense mechanisms to counter stressors like inflammation, damage, and free radicals during recovery. These enzyme changes, along with higher LDH levels, reflect physiological adjustments in response to stress, aligning with patterns observed in previous studies. This highlights the crucial role of muscle tissue in adapting to high-stress conditions and enhances our understanding of fish recovery processes [33].

CAT activity in the gill tissue significantly increased at high density. CAT helps to break down the common ROS hydroperoxides, thereby protecting cells from oxidative damage [18]. In the D1 and D2 groups, POD activity increased significantly during the initial stage of recovery, probably in response to moderate oxidative stress. POD activity in the D3 group increased significantly during the late recovery stage, suggesting a long-lasting defense reaction to continuous oxidative stress. These modifications imply that POD, a significant antioxidant enzyme, is crucial for fish to respond to oxidative stress [34]. A study on Totoaba found that changes in T-SOD activity were more pronounced in the muscle tissues than in the gills, and T-SOD activity has a significantly protective effect, particularly when combined with CAT activity [35]. T-SOD and LDH activities significantly increased under stress conditions, especially in the D2 group, which may be related to a balanced response between oxidative stress and energy demand. T-SOD protects cells from superoxide radicals, whereas LDH is involved in anaerobic glycolysis, suggesting that energy metabolism may change under stressful conditions owing to changing oxygen levels or physical limitations [36]. Malarvizhi showed that similar alterations in LDH activity adversely affected the overall health and physiological functions of common carp [37].

In this study, the activities of CAT, POD, T-SOD, and LDH in whole-body tissues of A. latus fry significantly increased under different density stresses, peaking in the later recovery stages. Notably, LDH activity reached its highest level during late recovery, indicating enhanced cellular metabolic activity, likely as an adaptation to initial stress. This result aligns with previous studies on metabolic changes in fish under stress conditions, highlighting LDH’s critical role in energy metabolism regulation and further validating the protective mechanism of antioxidant enzymes under oxidative stress [38,39].

HSP90, which consists mainly of the *HSP90α* and *HSP90β* isoforms, is a highly conserved family of proteins that is widely expressed in both prokaryotes and eukaryotes. They are predominantly found in the cytoplasm [40]. In the liver tissue, *HSP90α* expression levels were higher in the D3 group than in the other two density groups. Higher densities may lead to increased local environmental stresses, such as insufficient supply of nutrients and oxygen, resulting in the upregulation of *HSP90α* to help cells resist stress and maintain proper protein folding. This is consistent with the results reported by [39], who showed that exposure to density stress immediately induces a highly sensitive HSP90 phenotype. Oxygen-dependent HIF-1 consists of α and β subunits and is recognized as an important biomarker in fish, particularly *HIF-1α* [41]. *HIF-1α* expression levels in the liver tissue were not affected across density groups and recovery time points, which is consistent with the findings by Kwasek in perch. These expression patterns may be due to a species-specific response of *HIF-1α* expression to density stress. *IL-6* exerts pro- and anti-inflammatory effects [42]. It was initially identified as a B-cell stimulating factor that promotes the differentiation of B cells into plasma cells, leading to antibody production in vertebrates [43]. In the liver tissue, *IL-6* expression levels in the D1 group and the significant increase in *IL-6* in the early stages of recovery may reflect an acute inflammatory response to the initial stress. In contrast, the significant decrease in *IL-6* in the D2 and D3 groups in the later stages of recovery may indicate that the inflammatory response is controlled over time or that inflammatory signals diminish during tissue recovery. Both exogenous and endogenous apoptotic pathways activate the *caspase-3* gene, making it an important marker of the execution phase of the apoptotic pathway [44]. In the present study, the expression of *caspase-3* in liver tissues increased in the D1 and D2 groups with prolonged recovery time, whereas the expression decreased in the late stage of recovery in the D3 group, suggesting a possible synergistic effect with other genes [45].

The intestinal tissues had a persistently elevated expression of *HSP90α* and *HIF-1α*, indicating that these tissues could be under constant stress from environmental causes such as inflammation or hypoxia. This conclusion is consistent with an earlier study by Refaey, which found catfish to have higher levels of HSP90 during transportation [46]. This suggests that HSP90 may be a useful biomarker for stress in these situations. Furthermore, Adhikari showed that activation of *IL-6* influences an organism’s immunological response [47]. The significant decrease in *IL-6* expression in the D3 group at the later stages of recovery may suggest that the inflammation had subsided or that the intestinal cells had adapted to the initial inflammatory circumstances and stabilized the tissue through different means.

In muscle tissue, *HSP90α* expression levels in the D2 and D3 groups were relatively stable. This finding is consistent with that of a previous study on horse mackerel, in which no significant effects were observed [48]. These changes might be the result of variations across fish species. Hypoxic response factor *HIF-1α* typically exhibits elevated expression in hypoxic environments. The increase in *HIF-1α* expression in the D1 group during the pre-recovery phase would suggest that muscle cells in this group may have experienced hypoxia early in the recovery process, either as a result of an initial blood supply shortage or a high metabolic rate. The fish were subjected to eutrophication and reduced oxygen partial pressure in their aquatic habitat, which hindered the HIF-1 hydroxylation pathway and resulted in the buildup of HIF-1α protein as the duration of stress increased. Consequently, gene expression increased to adjust to the hypoxic environment [49]. This process is complex and may be connected to regeneration and repair processes. An essential component of the apoptotic process is *caspase-3*. As the recovery period increases, a percentage of the muscle cells may enter the apoptotic stage in response to the injury or as a method to eliminate damaged cells, as shown by the increase in *caspase-3* in the D1 group during the late stage of recovery in this study.

*HSP90α* gene expression in the gill tissues of the D1 group rose with recovery time, suggesting that these tissues were still under stress and that more *HSP90α* was required to assist the cells in withstanding the stress and preserving protein stability. The pattern of declining and then increasing *HSP90α* gene expression in the D2 and D3 groups may indicate how these tissues adapted to stress in the early stages of recovery and subsequently continued to do so or even improved under stress. These observations may be explained if density stress stimuli do not significantly affect the early heat stress response in the gill tissues, allowing environmental stimuli to reach a certain level of biotolerance before the intracellular RNA polymerase of HSPs is suddenly activated. This results in synergistic interactions with other proteins, which inhibit protein aggregation and repair heat denaturation [50,51]. Expression of *IL-6* increased in all density groups during the recovery phase, indicating that the inflammatory response grew stronger with time. This pattern may be associated with immune cell activation and the release of cytokines during tissue regeneration and repair. The expression of *caspase-3* did not change significantly after recovery. This could mean that the apoptotic process did not significantly increase under these circumstances or that the tissues had stabilized cell survival by adapting to the initial stress or injury. These results are consistent with those of Fu et al. (2020) for *Megalobrama amblycephala* and Cheng et al. (2017) for *M. amblycephala* under hypoxic stress [52,53].

*HSP90α* is considered important in many physiological processes linked to cell viability, chromosome stabilization, and stress management. In the whole-fish samples, expression of the *HSP90α* gene in the D4 group showed a decreasing trend, possibly indicating that cellular adaptation to the initial stress improved as the recovery proceeded or that the stressor had been removed or mitigated. In the D4 group, *HIF-1α* expression peaked 6 h after recovery and then declined after 12 and 18 h. The expression and transcriptional activity of *HIF-1α* have been demonstrated to be affected by the level of oxygen in the cells [54]. Geng et al. (2014) found that extended exposure to hypoxia resulted in a decrease in *HIF-1α* mRNA expression in spiny dogfish [55]. The observations in this study could indicate the occurrence of a temporary lack of oxygen or other metabolic stress, followed by a recovery in environmental or physiological conditions that resulted in a reduction in gene expression. This may be attributed to a feedback process that arises from the buildup of the HIF-1 protein [56]. Prior research conducted by Ton et al. (2003) and van der Meer et al. (2005) indicated that a decrease in *HIF-1α* expression was a result of hypoxic shock [57,58]. *IL-6* has been identified as a potential surrogate element in conjunction with other immune-related genes [59]. In the present study, we observed a significant reduction in the expression level of the *IL-6* gene in the D5 group after 18 h of recovery. This suggests that the inflammatory process was gradually controlled after its initial activation. This change may be linked to the activation of inflammatory mediators, such as the promotion of anti-inflammatory signaling or the relief of pathological conditions. Despite the presence of environmental or physiological stress, the gene expression level of *caspase-3* remained constant in all density groups and recovery time points, indicating that the apoptotic process was not significantly affected or that the apoptotic and regenerative processes were in a state of dynamic balance.

This study investigated the effects of transport stress at different densities on the activities of antioxidant enzymes and the expression of immune genes in various tissues of *A. latus* fry. The effects of different recovery periods after the end of transport on antioxidant enzyme activity and its related gene expression were assessed using a 12 h simulated transport assay. Our results indicate that density stress has a significant impact on the activities of antioxidant enzymes CAT, T-SOD, POD, and LDH in all tissues, with the effects being influenced by both density and recovery time. Specifically, antioxidant enzymes in the liver and intestinal tissues exhibited significant changes in activity, indicating a robust ability to adapt to oxidative stress. This adaptability was particularly evident in the high-density group, which exhibited a strong antioxidant response. In addition, gene expression of *HSP90α* and *caspase-3* also increased under high-density conditions, which may be related to the repair of damage and the activation of defense mechanisms in response to stress.

In summary, our findings provide key insights into the impact of density on physiological stress responses in fish larvae, which has significant implications for aquaculture practices aimed at optimizing transport conditions to enhance fish survival and health. This study aims to inspire further exploration and engagement from researchers and industry practitioners, contributing to the development of sustainable aquaculture practices that prioritize fish welfare.

## 5. Conclusions

We find that when plastic-sealed oxygen transport is not longer than 12 h, a bag containing 1200 tails of 1–2 cm *A. latus* fry or 125 tails of approximately 4–5 cm *A. latus* fry is safest. This study provides insights into the mechanisms underlying the effects of transport density on antioxidant and immune regulation in fry of *A. latus* and provides a scientific basis for fry transport management in aquaculture.

## Figures and Tables

**Table 1:** Primer sequences used in this experiment.

Primer	Primer Sequences (5′–3′)	Amplification Target
*HSP90α*-F	ACGACAAGGCTGTGAAGGAC	Expression of *HSP90α*
*HSP90α*-R	CTGTAGATGCGGTTGGAGTG	
*Caspase 3*-F	GCTGACTTCCTCTACGCTTT	Expression of *Caspase 3*
*Caspase 3*-R	AACTCTGTCGCCACCTTG	
*HIF-1α*-F	TGACAGAGGAGGGAGACA	Expression of *HIF-1α*
*HIF-1α*-R	TCACAGGGATGAACAAAGT	
*IL-6*-F	GTGCCTTCACTGACAATCC	Expression of *IL-6*
*IL-6*-R	ACTCCTGCCTGTGGGTCT	

## Data Availability

The original contributions presented in the study are included in the article/Supplementary Material, further inquiries can be directed to the corresponding authors.

## Supplementary Materials

The following supporting information can be downloaded at https://www.mdpi.com/article/10.3390/genes15111479/s1, Supplement S1. The mRNA levels of immune-related genes in 13 normal tissues.
