# Supplementary material for: Effects of Density Stress During Transportation on the Antioxidant Activity and Immuno-Related Gene Expression in Yellowfin Seabream (Acanthopagrus latus Houttuyn, 1782)
Source: Genes (Basel). 2024 Nov 17;15(11):1479. doi: 10.3390/genes15111479 (PMC11593578; doi:10.3390/genes15111479)

Supplement S1. The mRNA levels of immune-related genes in 13 normal tissues. *HIF-1α* (A), *HSP90α* (B), *Caspase 3* (C), *IL6* (D) expressions in liver, spleen, intestine, stomach, kidney, heart, brain, eye, muscle, skin, gills, fin, gonad of *A. latus* fry. The liver group was used as control.

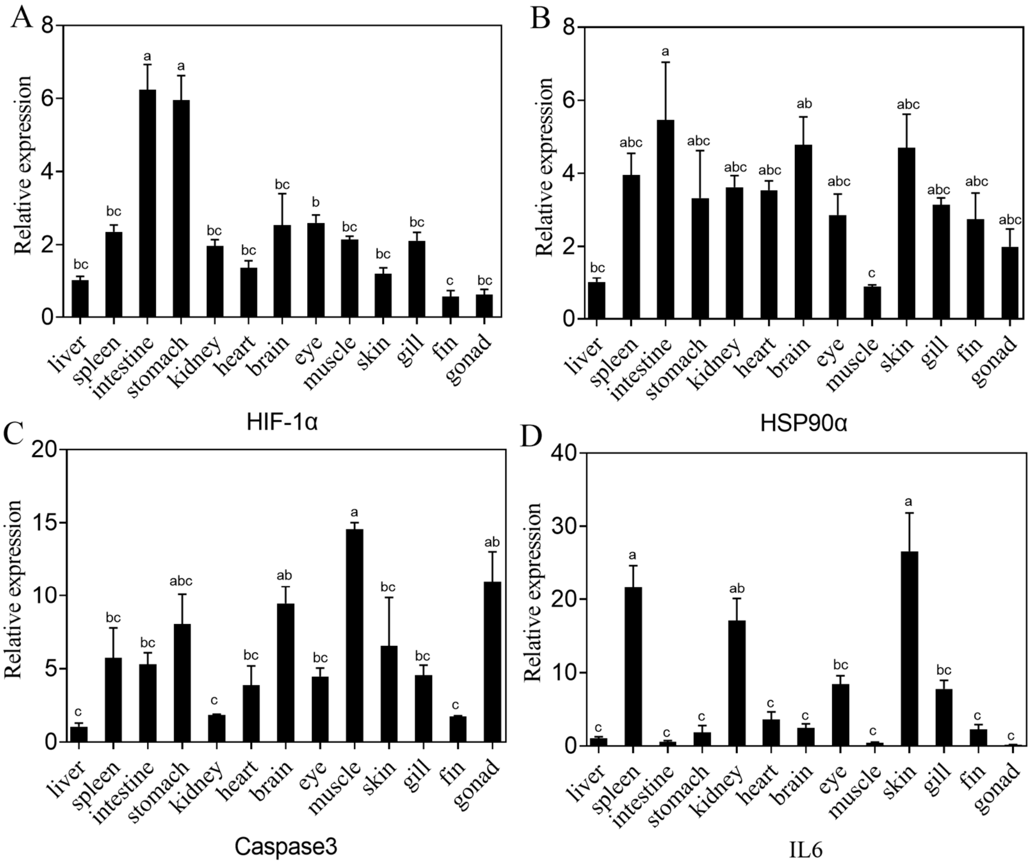

Supplement: Supplementary file 1 [file genes-15-01479-s001.zip › genes-3249594-supplementary.pdf]
